# Study on the cut-off point and the influencing factors of distress in newly diagnosed breast cancer patients

**DOI:** 10.3389/fpsyg.2024.1281469

**Published:** 2024-02-20

**Authors:** Ling Liu, Rong Wang, Yiming Sun, Ying Xiao, Guangsheng Du, Qingling Zhang

**Affiliations:** ^1^Department of General Surgery, The Second Affiliated Hospital of Army Medical University, Chongqing, China; ^2^Department of Medical Psychology, The Second Affiliated Hospital of Army Medical University, Chongqing, China

**Keywords:** distress thermometer, breast cancer, cut-off point, psychological screening tool, influencing factor

## Abstract

**Objective:**

Our aim is to investigate the cut-off point of distress and the influencing factors associated with distress in patients with newly diagnosed breast cancer.

**Methods:**

A cross-sectional survey of distress was conducted in 167 patients with newly diagnosed breast cancer admitted to the Department of General Surgery of a tertiary care hospital from July 2020 to March 2022. Patients completed the Hospital Anxiety and Depression Scale (HADS) and the Distress Thermometer (DT) questionnaire within 3 days of admission. The HADS ≥15 was used as the gold standard, and the cut-off point of the DT measure was analyzed using the Receiver Operating Characteristic (ROC) curve. The cut-off point obtained by ROC curve analysis was used to analyze the influencing factors of distress in breast cancer patients by univariate and multivariate regression analysis.

**Results:**

A total of 167 patients completed the survey, with an average HADS score of 8.43 ± 5.84 and a total HADS score of ≥15 in 37 (22.16%) patients, the mean DT score was 2.96 ± 1.85. ROC curve analysis showed an area under the curve of 0.885, with a maximum Jorden index (0.723) at a DT score of 4, the sensitivity was 100.0% and specificity was 72.3%. There were 73 (43.71%) patients with DT score ≥ 4. Regression analysis showed that insurance/financial problems, dealing with partner problems, tension, bathing/dressing problems, pain, and sleep problems were independent risk factors for l distress in newly diagnosed breast cancer patients.

**Conclusion:**

A DT score 4 is the cut-off point for distress in patients with newly diagnosed breast cancer. In clinical practice, target intervention should be carried out according to the risk factors of distress of patients.

## 1 Introduction

Breast cancer, the most common type of tumor in women, surpassed lung cancer in 2020 to become the most prolific cancer in the world at 11.7% of all cancers, according to Global Cancer Statistics 2020 (GLOBCAN) published online by *CA: A Cancer Journal for Clinicians* in 2021(11.7%) (Sung et al., 2021). Among women, breast cancer is the leading cause of morbidity (24.5%) and mortality (15.5%) (Sung et al., 2021). Patients with breast cancer generally face various physical and psychological problems during diagnosis and treatment of the disease, including pain, fatigue, sleep disorders, anxiety, depression, perceived stress, cognitive dysfunction, and fear of recurrence (Lengacher et al., 2012), which seriously affect their quality of life (QOL). In recent years, the National Comprehensive Cancer Network (NCCN) has continuously updated the NCCN clinical guidelines for cancer, in which the word “distress” benefits cancer patients reducing the shame of psychological problems and improving the acceptance of cancer patients and doctors in psychological problems (Qi et al., 2018).

Distress is an emotional experience caused by psychological (cognitive, behavioral, emotional), social and/or spiritual experiences, which manifests as emotional reactions such as vulnerability, sadness, fear or serious psychological problems such as depression, anxiety, fear, feelings of social isolation and severe psychological problems (Stanton and Bower, 2015). The incidence of distress in patients with breast cancer is 25.3–71.7% (Iskandarsyah et al., 2013; Ploos van Amstel et al., 2013; Gibbons et al., 2016; Sun et al., 2021). Studies have shown that long-term negative emotions are an important cause of breast cancer, and the treatment process may increase anxiety and depression due to treatment, worry and fear of cancer death and recurrence; and the lack of improvement in long-term psychological distress may even increase mortality (Schoepf and Heun, 2015; Stanton and Bower, 2015). Therefore, it is particularly important to accurately identify the distress of breast cancer patients in a timely manner.

There are many clinical tools used to screen for distress, including the General Distress Scale, the Tumor-Related Symptoms Scale, the Psychiatric Symptoms Scale, the Quality of Life and Somatic Functioning Scale, and the Patient Needs and Social Reality Scale (Zhang and Tang, 2017). Most scales have many items and complex evaluations. Evaluators need to receive professional training, which is not suitable for preliminary screening (Zhang and Tang, 2017). The Distress Thermometer (DT) was developed by Roth et al. (1998) and first applied to patients with prostate cancer. The National Comprehensive Cancer Network (NCCN) added the Problem List (PL) to the DT and first published guidelines for the management of distress in 1999. This guideline recommends the use of the DT for screening distress levels and associated factors (Jacobsen and Ransom, 2007; Jacobsen et al., 2011; Donovan and Jacobsen, 2013). The DT was translated into Chinese by Zhang Yening in 2010, and its reliability and validity were studied in patients with cancer in China. The total Cronbach’s α coefficient of the scale was 0.948 (Wei et al., 2021). The Hospital Anxiety and Depression Scale (HADS) was created by Zigmond and Snaith (1983) in 1983 and is one of the most commonly used tools for screening anxiety and depression in physical diseases (Julian, 2011; Stern, 2014). It has been used as the gold standard in several studies (Ransom et al., 2006; Shim et al., 2008; Baken and Woolley, 2011; Bidstrup et al., 2012). DT, as a distress screening tool recommended by NCCN, has fewer items than HADS and is easy to administer.

Although domestic and foreign studies (Ng et al., 2017; Admiraal et al., 2019; Liping et al., 2019; Civilotti et al., 2020) have used DT to screen for distress among breast cancer patients and verified the accuracy and effectiveness of the DT, there are few studies on the related factors influencing distress among breast cancer patients, and the cut-off points of the DT differs from state to state. This study aimed to explore the cut-off point of the DT and the related factors influencing distress in newly diagnosed breast cancer patients.

## 2 Materials and methods

### 2.1 Participants

A total of 167 female patients newly diagnosed with breast cancer in the Department of General Surgery of our hospital from July 2020 to March 2021 were selected as the research subjects. The inclusion criteria were as follows: (1) age ≥ 18 years, (2) pathological or cytological diagnosis of breast cancer and first admission, (3) female sex, (4) anonymity, (5) ability to read and write Chinese (elementary school education or more), and (6) consent to participate in this study. The exclusion criteria were as follows: (1) patients with a previous history of psychiatric disorders, hearing impairment or communication impairment who were unable to complete the questionnaire; (2) those who had received psychological counseling or psychotherapy within the previous 3 months; and (3) those who were taking anti-anxiety and depression medications. This study met the ethical requirements and was reviewed and approved by the hospital ethics committee (2020-Research No.021–01) and registered in the National Clinical Trials Registry online.

### 2.2 Methods

#### 2.2.1 Survey instruments

(1) General information questionnaire: The main contents included height, weight, gender, date of birth, ethnicity, occupation, education level, marital status, spouse’s education level, family per capita income and sleep quality (poor, general, good). (2) HADS (Zigmond and Snaith, 1983): The HADS consists of two subscales, the anxiety subscale HADS-A and the depression subscale HADS-D. Each scale consists of seven entries, and each item is scored from 0 to 3. The total scale score of the HADS-T is the sum of the HADS-D and HADS-A subscale scores. A subscale score < 8 indicates no symptoms, 8–11 indicates suspected symptoms, and > 11 indicates the definite presence of anxiety and depression. In this study, a total score ≥ 15 on the two subscales was used as the diagnostic criterion (Julian, 2011). (3) Distress Thermometer (DT): The DT is a self-assessment tool that assesses the patient’s level of distress in the previous week, on a scale of 0–10, with 0 indicating no distress and 10 indicating extreme distress. The PL includes five types of practical problems, family problems, emotional problems, physical problems, and spiritual/religious concerns, with a total of 39 items. If there are problems that do not belong to these five types of problems, they are defined as other problems and counted as 1 item, making a total of 40 entries.

#### 2.2.2 Study methods

The researchers were specially trained, and all subjects gave informed consent for the study before completing the questionnaire. All assessments were completed within 3 days of patient admission.

#### 2.2.3 Statistical methods

All data were processed using SPSS 25.0 statistical software. Taking HADS diagnosis results as the gold standard, a receiver operating characteristic curve (ROC) was used to test the diagnostic accuracy of DT compared with HADS, and the area under the curve (AUC) and Jorden index were calculated. The measurement data are expressed as the mean ± standard deviation ( $\bar{x}$ ±S), and an independent samples t test was used for comparisons between groups. Count data were expressed as frequencies or percentages (%), and comparisons were made using the χ^2^-test or Fisher’s exact probability method. Logistic regression analysis was used for multifactorial analysis, stepwise regression methods were also applied, and significant differences were indicated by *p* < 0.05.

## 3 Results

### 3.1 Diagnostic efficacy of DT for distress

**Figure 1:**
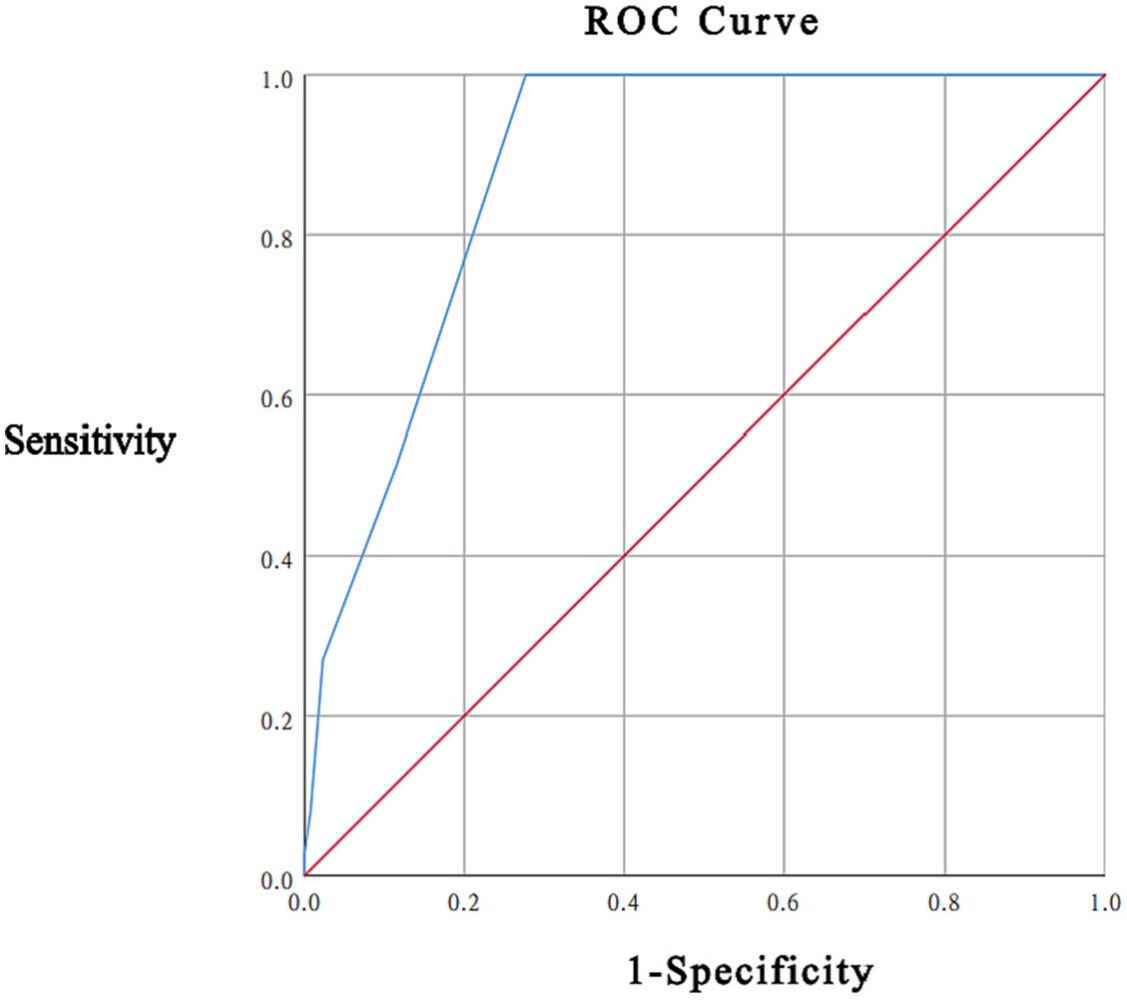
ROC graph of DT distress thermometer score.

In this study, 37 (22.16%) participants had a total HADS score ≥ 15, with a mean HADS score of 8.43 ± 5.84; the mean DT score was 2.96 ± 1.85. As recommended by the NCCN guidelines, a DT score < 4 is considered low distress, 4 ≤ DT score ≤ 6 is considered moderate distress, and a DT score ≥ 7 is considered high distress. In this study, 73 patients (43.71%) had a DT score ≥ 4 points, including 69 patients with a DT score of 4–6 points and 4 patients with a DT score ≥ 7 points. ROC curve analysis took the total HADS score ≥ 15 as the gold standard, and the area under the curve (AUC) was 0.885 (95% CI was 0.836–0.933, *p* < 0.001). When the threshold of the DT score was 4 points, the sensitivity was 100.0%, the specificity was 72.3%, and the maximum Youden index was 0.723 (see Figure 1).

### 3.2 Survey of distress problems among newly diagnosed breast cancer patients

**Figure 2:**
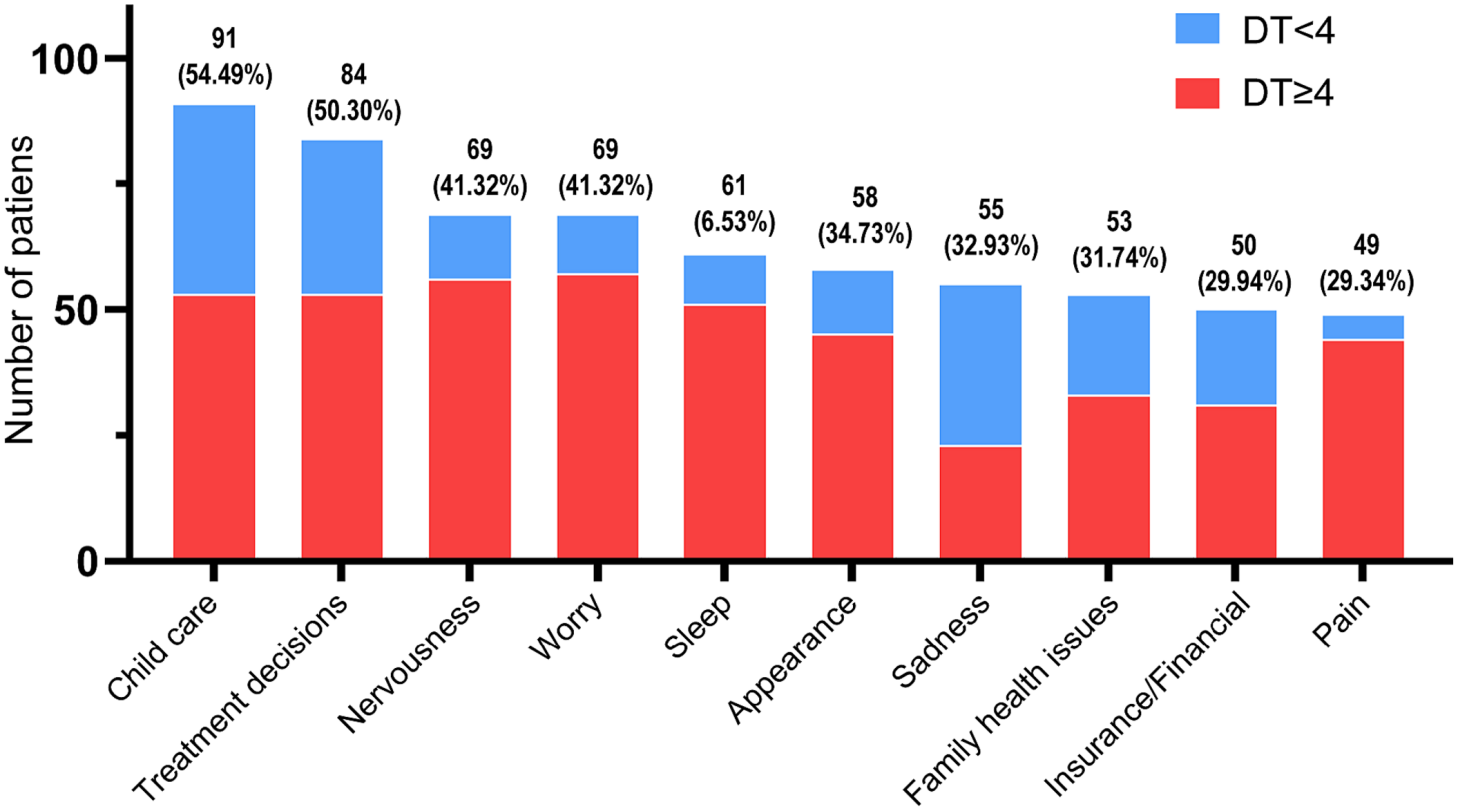
Top 10 distribution of DT problem list.

For statistical analysis of the PL, the top 10 problems were 91 (54.49%) child care, 84 (50.30%) treatment decisions, 69 (41.32%) nervousness, 69 (41.32%) worry, 61 (36.53%) sleep, 58 (34.73%) appearance, 55 (32.93%) sadness, 53 (31.74%) family health issues, 50 (29.94%) insurance/financial, and 49 (29.34%) pain (see Figure 2).

### 3.3 General information analysis of distress in newly diagnosed breast cancer patients

Based on the above statistics, the DT cut-off point of 4 was used as the basis for grouping the study participants into two groups to analyze the impact of general information on the distress of patients with newly diagnosed breast cancer. The results showed that there were significant differences in age and monthly household income between the two groups (*p* < 0.05) (see Table 1).

**Table 1:** Single-factor analysis of influencing demographic characteristics of distress in newly diagnosed breast cancer patients [*n* (%), $\bar{x}\pm s$ ].

Parameters	DT<4 (*n* = 94)	DT ≥ 4 (*n* = 73)	χ^2^/t	*p*
Age (years)	50.76 ± 7.82	46.90 ± 8.59	3.040	0.003
Education level			2.116	0.347
Junior high school and below	71(75.50)	51(69.90)		
High school and special (or technical) secondary school	20(21.30)	16(21.90)		
Junior college and above	3(3.20)	6(8.20)		
Career			3.151	0.207
Workers	39(41.50)	40(54.80)		
Farmers	14(14.90)	10(13.70)		
Other	41(43.60)	23(31.50)		
Marital status			0.677	0.411
Married	86(91.50)	70(95.90)		
Unmarried/divorced/widowed	8(8.50)	3(4.10)		
Monthly household income			6.697	0.035
<3,500	19(20.20)	12(16.40)		
3,500–6,000	64(68.10)	41(56.20)		
>6,000	11(11.70)	20(27.40)		

### 3.4 Analysis of PL-related factors in distress

Similarly, based on the DT cut-off point of 4, the research subjects were divided into two groups to analyze the impact of PL-related factors on the distress of patients with newly diagnosed breast cancer. Because the PL of the two groups was not involved in housing, breathing, changes in urination, diarrhea, feeling swollen, mouth sores and substance use, the above items were excluded. There were statistically significant differences in child care, insurance/financial, treatment decisions, dealing with children, dealing with partner, family health issues, fears, nervousness, worry, appearance, bathing/dressing, indigestion, memory/concentration, nausea, pain and sleep between the two groups (*p* < 0.05) (see Table 2).

**Table 2:** A univariate analysis of DT problem list affecting distress.

Parameters	Options	DT<4 (*n* = 94)	DT ≥ 4 (*n* = 73)	χ^2^/t	*p*
Child care	Yes	38(40.40)	53(72.60)	17.156	<0.001
	No	56(59.60)	20(27.40)		
Insurance/financial	Yes	19(20.20)	31(42.50)	9.700	0.002
	No	75(79.80)	42(57.70)		
Transportation	Yes	1(1.10)	2(2.70)	0.049	0.825
	No	93(98.90)	71(97.30)		
Work/school	Yes	13(13.80)	15(20.50)	1.329	0.249
	No	81(86.20)	58(79.50)		
Treatment decisions	Yes	31(33.00)	53(72.60)	25.806	<0.001
	No	63(67.00)	20(27.40)		
Dealing with children	Yes	7(7.40)	18(24.70)	9.562	0.002
	No	87(92.60)	55(75.30)		
Dealing with partner	Yes	2(2.10)	8(11.00)	4.232	0.040
	No	92(97.90)	65(89.00)		
Ability of have children	Yes	11(11.70)	2(2.70)	3.434	0.064
	No	83(88.30)	71(97.30)		
Family health issues	Yes	20(21.30)	33(45.20)	10.860	0.001
	No	74(78.70)	40(54.80)		
Depression	Yes	3(3.20)	4(5.50)	0.117	0.732
	No	91(96.80)	69(94.50)		
Fears	Yes	5(5.30)	25(34.20)	23.332	<0.001
	No	89(94.70)	48(65.80)		
Nervousness	Yes	13(13.80)	56(76.70)	67.012	<0.001
	No	81(86.20)	17(23.30)		
Sadness	Yes	32(34.00)	23(31.50)	0.120	0.729
	No	62(66.00)	50(68.50)		
Worry	Yes	12(12.80)	57(78.10)	72.299	<0.001
	No	82(87.20)	16(21.90)		
Loss of interest in usual activities	Yes	2(2.10)	2(2.70)	0.000	1.000
	No	92(97.90)	71(97.30)		
Spiritual/religions concerns	Yes	1(1.10)	1(1.40)	0.000	1.000
	No	93(98.90)	72(98.60)		
Appearance	Yes	13(13.80)	45(61.60)	41.440	<0.001
	No	81(86.20)	28(38.40)		
Bathing/dressing	Yes	2(2.10)	23(31.50)	25.602	<0.001
	No	92(97.90)	50(68.50)		
Parameters	Options	DT<4 (*n* = 94)	DT ≥ 4 (*n* = 73)	χ^2^/t	*p*
Breathing	Yes	3(3.20)	5(6.80)	0.537	0.464
	No	91(96.80)	68(93.20)		
Eating	Yes	0(0.00)	2(2.70)	Fisher	0.190
	No	94(100.00)	71(97.30)		
Feeling swollen	Yes	2(2.10)	3(4.10)	0.083	0.774
	No	92(97.90)	70(95.90)		
Fevers	Yes	0(0.00)	3(4.10)	Fisher	0.082
	No	94(100.00)	70(95.90)		
Getting around	Yes	2(2.10)	0(0.00)	Fisher	0.505
	No	92(97.90)	73(100.0)		
Indigestion	Yes	1(1.10)	7(9.60)	4.812	0.028
	No	93(98.90)	66(90.40)		
Memory/congested	Yes	2(2.10)	8(11.10)	4.232	0.040
	No	92(97.90)	65(89.00)		
Nausea	Yes	0(0.00)	4(5.50)	Fisher	0.035
	No	94(100.00)	69(94.50)		
Nose dry/congested	Yes	2(2.10)	1(1.40)	0.000	1.000
	No	92(97.90)	72(98.60)		
Pain	Yes	5(5.30)	44(60.30)	59.855	<0.001
	No	89(94.70)	29(39.70)		
Sexual	Yes	0(0.00)	3(4.10)	Fisher	0.082
	No	94(100.00)	70(95.90)		
Skin dry/itchy	Yes	7(7.40)	3(4.10)	0.328	0.567
	No	87(92.60)	70(95.90)		
Sleep	Yes	10(10.60)	51(69.90)	62.164	<0.001
	No	84(89.40)	22(30.10)		
Tingling in hands/feet	Yes	0(0.00)	2(2.70)	Fisher	0.190
	No	94(100.00)	71(97.30)		

Using the presence or absence of distress as the dependent variable (DT < 4 points assigned 0, DT ≥ 4 points assigned 1), a multivariate logistic regression equation was constructed by including age, monthly household income, child care, insurance/financial status, treatment decisions, dealing with children, dealing with partner, family health issues, fears, nervousness, worry, appearance, bathing/dressing, indigestion, memory/concentration, nausea, pain and sleep. A total of six items were included in the final model by the stepwise regression method. The results showed that dealing with partner (OR = 31.831, 95% CI 3.929 ~ 257.887, *p* = 0.001), pain (OR = 11.048, 95% CI 2.683–45.498, *p* = 0.001), bathing/dressing (OR = 10.613, 95% CI 1.487–75.742, *p* = 0.018), sleep (OR = 4.434, 95% CI 1.169–16.811, *p* = 0.029), nervousness (OR = 4.253, 95% CI 1.065–16.985, *p* = 0.040) and insurance/financial issues (OR = 4.235, 95% CI 1.317 ~ 13.614, *p* = 0.015) were independent risk factors for increasing distress (see Table 3).

**Table 3:** Logistic regression analysis of independent risk factors for the occurrence of distress.

Parameters	*B*-value	*B*-value Standard error	Wald χ^2^	*p*-value	Exp (B)	Exp (B) 95% CI	
						Lower limit	Upper limit
Dealing with partner	3.460	1.067	10.510	0.001	31.831	3.929	257.887
Pain	2.402	0.722	11.066	0.001	11.048	2.683	45.498
Bathing/Dressing	2.362	1.003	5.549	0.018	10.613	1.487	75.742
Sleep	1.489	0.680	4.796	0.029	4.434	1.169	16.811
Nervousness	1.448	0.707	4.198	0.040	4.253	1.065	16.985
Insurance/financial	1.443	0.596	5.869	0.015	4.235	1.317	13.614

## 4 Discussion

### 4.1 The cut-off point of distress in newly diagnosed breast cancer patients

The ROC analysis showed that a DT score of 4 was the cut-off point to identify psychological distress in newly diagnosed breast cancer patients. The DT threshold of this study was 4 points, which is higher than the DT threshold of 2 points versus 3 points reported by Bidstrup et al. (2012). in Denmark for patients with primary breast cancer. Iskandarsyah et al. (2013) reported an optimal 5 points in Indonesia for patients with breast cancer, and Floortje K (Ploos van Amstel et al., 2017) reported an cut-off point of 7 points for the initial diagnosis of breast cancer in the Netherlands. This was close to the DT threshold of 4 points versus 5 points reported by Civilotti et al. (2020) for newly diagnosed Italian breast cancer patients and the same as the DT threshold reported by Alosaimi et al. (2018) for Saudi cancer patients, Sun et al. (2021) for Asian cancer patients, and Nguyen et al. (2021) for Vietnamese cancer patients. At the same time, it is also consistent with the DT threshold of 4 points recommended by the US National Cancer Network for cancer patients (National Comprehensive Network Cancer Network, 2013). The different DT thresholds of breast cancer patients reported in the literature may be related to the differences in region, culture, cognitive level, sample size, disease cycle, and treatment status. According to the literature (Iskandarsyah et al., 2013; Ploos van Amstel et al., 2013; Gibbons et al., 2016), the incidence of distress in breast cancer patients is 41–52%. In a systematic review and meta-analysis by Sun et al. (2021), 3,870 breast cancer patients were included, and the incidence of distress was 25.3–71.7%. In this study, using the DT score of 4 as the threshold, the incidence of distress in breast cancer patients was 43.71%, which was higher than the 22.16% obtained using the HADS total score of 15 as the screening standard. This can make us more vigilant and help us take effective interventions before distress becomes harmful to patients. A good screening tool should have sensitivity, specificity, stability, practicality, safety, simplicity and economy. DTs have the above characteristics and can be used in clinical work to quickly and effectively identify the distress of breast cancer patients.

### 4.2 Influencing factors of distress in newly diagnosed breast cancer patients

Insurance/financial distress is an important practical issue affecting the psychological distress of newly diagnosed breast cancer patients. In a study by Ying et al. (2019), economic problems ranked first. In this study, insurance/financial status ranked ninth in PL (29.94%), and multivariate analysis also confirmed that insurance/financial status was an independent risk factor affecting distress. This is consistent with the results of Rocque et al. (2016). This is mainly because cancer treatment requires financial support, and a good family economic situation and the purchase of insurance are the greatest protection against the economic burden of cancer treatment. In China, the reimbursement rate varies among different insurance policies, and the reimbursement rate of commercial insurance is higher than medical insurance. However, due to economic restrictions and conservative thinking, most patients do not purchase commercial insurance, and some patients do not purchase medical insurance either. In the treatment of breast cancer, some targeted drugs are expensive, and medical insurance does not cover the cost or the reimbursement ratio is low. If insurance is not purchased and the family’s economic situation is poor, the treatment of patients will be directly affected. Therefore, the lack of insurance combined with a poor economic situation will increase the distress of breast cancer patients. Health care workers should pay more attention to patients without insurance and those with a poor economic condition when caring for their psychological needs.

The problem of dealing with partners is a family problem that affects the distress of newly diagnosed breast cancer patients. Breasts, as a female secondary sexual characteristic, are often considered a symbol of female beauty. It is also positioned as an important part of a woman’s sense of self, so much so that mastectomy is associated with “half a woman” (Langellier and Sullivan, 1998). Some patients worry that after mastectomy, asymmetric breasts will affect their intimate relationship with their partner and even the quality of their sexual life. Studies have shown that low spousal support for breast cancer patients can predict high psychological stress and reduced QOL (Lewis et al., 2001). Spousal relationships and partner support are also deeply affected by breast cancer treatment (Bloom et al., 1998; Sandgren et al., 2004), even long after treatment ends (Bloom et al., 2004). Good couple relationships and supportive intimate partners have been shown to help women cope better with cancer (Ganz et al., 1999).

Nervousness is an emotional problem that affects the distress of newly diagnosed breast cancer patients. Nervousness ranked third in PL (41.32%) in this study, which was lower than that reported by Wenqian et al. (2020). It was second in PL for patients undergoing chemotherapy, which was higher than that reported by Ying et al. (2019), who ranked it fourth on the PL at the diagnosis stage. This may be related to the fact that the patients in this study all completed the questionnaire within 3 days of admission to the hospital with newly diagnosed breast cancer. Newly diagnosed breast cancer is a serious psychological stress event (Ying et al., 2019). At the same time, facing surgery, perhaps the loss of a breast, is difficult for patients to accept and inevitably produces nervousness. In addition, female patients are more likely to worry about changes in body shape after mastectomy, weight gain or loss, and their partners’ difficulty in accepting their body shape changes, which may aggravate their negative emotions (Archibald et al., 2006).

Bathing/dressing problems, sleep problems and pain were the physical problems that affected the distress levels of newly diagnosed breast cancer patients. Syrowatka et al. (2017) showed that pain and sleep disorders were related to distress in breast cancer patients. Bjerkeset et al. (2020) also showed that there was a correlation between pain and breast cancer distress. Alosaimi et al. (2018) reported that pain and sleep are independent factors of distress in cancer patients. As the fifth vital sign, pain seriously affects the QOL of patients. Breast cancer and surgery can cause pain. Preoperative worry about postoperative pain and the discomfort caused by postoperative pain will directly increase the distress of patients. Studies have reported (Dabrowski et al., 2007) that the incidence of sleep disorders in cancer patients is between 25 and 59%, but this problem is rarely found or resolved in cancer treatment. Sleep disorders can lead to poor QOL and treatment intolerance, and severe cases can develop into depression (Stanton, 2012), which further aggravates the distress of patients. Chan et al. (2018) reported that bathing/dressing and dressing difficulties were the most common problems associated with distress. This was also demonstrated in this study. This may be related to the patient’s concern about the decline in self-care ability after surgery and the limitation of limb movement on the affected side, which may affect bathing/dressing. In clinical practice, we find that this concern is more obvious in patients with right-sided breast cancer.

Because this study is a small observational study with a small sample size, it was completed within 3 days of newly diagnosed breast cancer. Therefore, the results of this study have certain limitations and may not reflect the dynamic changes in patients’ distress. Further studies with larger sample sizes are needed.

## 5 Conclusion

In conclusion, a DT score of 4 is the cut-off point for distress in newly diagnosed breast cancer patients. The sources of distress for breast cancer patients vary. The use of DT and PL can provide an understanding of the degree of distress for patients and the related factors influencing distress, which is simple, clear and easy to administer. In clinical work, medical workers should pay attention to the assessment of distress among breast cancer patients and take corresponding intervention measures according to the assessment results to reduce patients’ degree of distress, promote their physical and mental health, and improve their QOL.

## Data availability statement

The original contributions presented in the study are included in the article/supplementary material, further inquiries can be directed to the corresponding authors.

## Ethics statement

The studies involving humans were approved by the Second Affiliated Hospital of Army Medical University of PLA Medical Ethical Committee. The studies were conducted in accordance with the local legislation and institutional requirements. The participants provided their written informed consent to participate in this study.

## Author contributions

LL: Writing – original draft, Writing – review & editing. RW: Data curation, Investigation, Writing – original draft. YS: Software, Supervision, Writing – review & editing. YX: Investigation, Writing – original draft. GD: Funding acquisition, Writing – review & editing. QZ: Conceptualization, Funding acquisition, Investigation, Writing – review & editing.
